# Quality of tuberculosis care by pharmacies in low- and middle-income countries: Gaps and opportunities

**DOI:** 10.1016/j.jctube.2019.100135

**Published:** 2019-12-02

**Authors:** Rosalind Miller, Catherine Goodman

**Affiliations:** Department of Global Health and Development, London School of Hygiene and Tropical Medicine, 15-17 Tavistock Place, London, WC1H 9SH, United Kingdom

**Keywords:** Pharmacy, Tuberculosis, Private sector, Drug shops, Quality of care

## Abstract

Pharmacies hold great potential to contribute meaningfully to tuberculosis (TB) control efforts, given their accessibility and extensive utilisation by communities in many high burden countries. Despite this promise, the quality of care provided by pharmacies in these settings for a range of conditions has historically been poor. This paper sets out to conceptualise the key issues surrounding quality of TB care in the low- and middle-income country pharmacy setting; examine the empirical evidence on quality of care; and review the interventions employed to improve this. A number of quality challenges are apparent in relation to anti-TB medicine availability, pharmacopeial quality of anti-TB medicines stocked, pharmacy workers’ knowledge, and management of patients both prior to and following diagnosis. Poor management practices include inadequate questioning of symptomatic patients, lack of referral for testing, over-the-counter sale of anti-TB medication as well as unnecessary and harmful medicines (e.g., antibiotics and steroids), and insufficient counselling. Interventions to improve pharmacy practice in relation to TB control have all fallen under the umbrella of public-private mix (PPM) initiatives, whereby pharmacies are engaged into national TB programmes to improve case detection. These interventions all involved training of pharmacists to refer symptomatic patients for testing and have enjoyed reasonable success, although achieving scale remains a challenge. Future interventions would do well to expand their focus beyond case detection to also improve counselling of patients and inappropriate medicine sales. The lack of pharmacy-specific global guidelines and the regulatory environment were identified as key areas for future attention.

## 1 Introduction

In low- and middle-income countries (LMICs), the core function of community pharmacists has long been narrowly focused on dispensing and retailing. In the context of tuberculosis (TB) care, this limits the role of pharmacies to filling prescriptions for anti-TB medication. However, their potential importance in the fight against TB could be so much greater, given their accessibility and extensive utilisation by communities. First, pharmacists can play a key role in diagnosis. Research from many high burden countries reports that patients experiencing the non-specific symptoms of TB (typically a prolonged cough) often seek care, in the first instance, at a private pharmacy [1], [2], [3], [4]. Poor quality management at this stage can lead to delayed diagnosis and negatively affect both case detection and disease control. Capitalising on diagnostic opportunities in pharmacy settings is pertinent given that 3.6 million (36%) of TB cases globally are deemed to be ‘missing’ [5] and the top three countries accounting for this global gap (India, Nigeria and Indonesia) all have large community pharmacy sectors. For illustration, Indonesia has 24,716 pharmacies which account for 52% of initial care seeking, yet currently notify not a single case [6]. Secondly, pharmacists have a key role in managing TB patients following diagnosis; this involves ensuring that anti-TB medications are dispensed (against prescriptions) alongside appropriate advice to ensure safe and effective use, and encourage adherence. Finally, given their prevalence in many high burden settings, pharmacies could take on a role in the provision of directly observed therapy (DOTS), a cornerstone of the Stop TB Strategy [7].

The importance of pharmacies for TB control efforts has been recognised at the global level and this falls within a wider movement to encourage the engagement of private providers within national TB programmes (public private mix- PPM) [8]. A joint statement from the International Pharmaceutical Federation (FIP) and the World Health Organization (WHO) in 2011 urged ‘national TB programmes and national pharmacy associations to develop and implement plans for engaging pharmacists in the fight against TB’ [9]. Konduri and colleagues have shown, however, that this recommendation has not been widely translated into practice, and pharmacy involvement remains nominal [10].

Despite the promise that LMIC pharmacies hold for global TB control efforts, the quality of care they provide across a range of health conditions has, historically, been plagued by shortcomings. Across Africa, Asia and Latin America research has consistently highlighted frequent absence of trained pharmacists or other qualified personnel; poor practices in filling prescriptions; inadequate management of patients including insufficient history taking, lack of appropriate medical referral, sale of medicines that are clinically inappropriate, and limited provision of advice; and endemic regulatory infringement such as sale of prescription only medicines (POMs) without a prescription [11,12].

This paper explores the potential of pharmacies to address these well-documented quality shortfalls in relation to TB care. It forms part of a series of papers examining various aspects of quality in relation to TB control [13]. We define ‘pharmacy’ in a broad sense - that is any outlet or individual who is recognised by the regulatory system to sell medicines. This includes licensed pharmacies, whether or not a qualified pharmacist is actually present, and also in some countries authorised drug shops where non-pharmacist personnel are allowed to operate a basic pharmacy, for example Accredited Drug Dispensing Outlets (ADDOs) in Tanzania or Patent Medicine Vendors (PMVs) in Nigeria. We begin by conceptualising the key issues surrounding quality of TB care in the pharmacy setting. Secondly, we examine the methods used for studying quality of care in pharmacies. Thirdly, we provide an overview of the empirical evidence on the quality of TB care provided by pharmacies in LMICs, and the interventions employed to improve this. Finally, we reflect on the future of pharmacy involvement in TB control efforts.

## 2 What quality of care do we expect from pharmacies?

Quality can be considered to have three broad dimensions: structure or foundations, processes and outcomes [23,24]. We focus mainly on the process of care, given that this element is directly in the hands of pharmacy workers. While structural quality has been cited as a poor proxy for the process of diagnosis and treatment [25], [26], [27], we also consider some essential structural elements (workforce and tools) (Table 1). Our focus is primarily on these technical aspects of care provision, rather than user experience.

**Table 1:** Key aspects of TB care quality in pharmacies.

Structural aspects/foundations of quality	Processes of care
• Knowledge of pharmacy staff regarding TB care for symptomatic and diagnosed patients. • Availability of anti-TB medicines. • Pharmacopeial quality of anti-TB medicines.	• Appropriate referral of symptomatic, undiagnosed TB patients presenting at the pharmacy. • Ensuring that symptomatic, undiagnosed TB patients do not receive harmful medicines, such as antibiotics. • Accuracy of dispensing of prescriptions for anti-TB medicines. • Quality of counselling to accompany dispensing of anti-TB medicines.

**Table 2:** Examples of TB case presentations and management of TB in standardised patient studies.

Study	Case presentation	Management	
		Referred	Sold an antibiotic
Miller and Goodman 2017, India [40]	‘I have had cough and some fever for 3–4 weeks. We have had a relative staying with us who has TB. Can you suggest something?’	46%	16%
Satyanarayana et al. 2016 (case 1), India [30]	‘I am having cough for nearly a month now and also have fever.' Whilst showing a positive sputum report to the chemist, the patient continues, 'I went to the government dispensary and they asked me to get my sputum tested. I have this report. Can you please give me some medicine?'	16%	37%
Satyanarayana et al. 2016 (case 2), India [30]	`I have cough and fever that is not getting better. Please give me some medicine.'	67%	16%
Vu et al. 2012, Vietnam [41]	SP claimed to be suffering from cough and fever for 4 weeks. No improvement had occurred after two 10-day courses of antibiotics (amoxicillin followed by spiramycin). SP had been in contact with a TB patient. Anti-TB drugs were requested. (The paper does not provide the verbatim script).	46%	41%

Whilst the WHO's ‘International Standards for TB Care’ provides general guidance for all providers on the diagnosis and treatment of TB internationally, there is no single global document available that covers the pharmacy's role in TB care holistically. It can thus be difficult to benchmark the quality of current practices. However, there are some national pharmacy-specific TB guidelines. For example, the Revised National Tuberculosis Control Programme (RNTCP), India, released a ‘training module for community pharmacists’ in collaboration with the Indian Pharmacy Association which sets out the role and responsibilities of the pharmacist in TB care [28]. These guidelines are explicit in terms of the pharmacist's role under headings such as ‘case detection and referral of TB suspects’ and ‘rational use of antibiotics and anti-TB drugs’. Further, there are sections of global level documents that are relevant e.g., WHO's ‘Practical Pharmacy For Developing Countries’ issue on TB and FIP/WHO's joint statement on the role of pharmacists in TB care [9,29]. Drawing on these, Box 1 outlines what pharmacy workers arguably should be doing in relation to diagnosis, dispensing, and counselling.

Box 1 **Expectations of pharmacy workers in TB management**

**Diagnosis**

The management of a TB patient is outside the realm of a pharmacist's expertise and, as such, suspected cases should be referred for testing. Some national programmes (or pilot projects) encourage pharmacies to actively triage and refer patients with a cough of duration two weeks or longer for sputum testing to a nearby designated microscopy centre. First or second line anti-TB medicines, along with any antibiotics (especially fluoroquinolones) should not be sold without a prescription. Steroids can mask TB symptoms leading to a delayed diagnosis and thus should not be sold over-the-counter (OTC) [30].

**Dispensing**

One area where the pharmacist's role is clear-cut is in dispensing of anti-TB medicines. These medicines should be dispensed in accordance with the prescription, in the correct dosage, for the specified duration. FIP/WHO specify that pharmacists should ‘ensure that quality-assured medicines are procured and supplied and that fixed-dose combinations recommended by WHO are used. Furthermore, dispensing anti-TB medicines that have not been certified as safe and effective and sale of inappropriate combinations should be stopped’ [9]. Where pharmacists detect potential errors on prescriptions for TB medicines, the prescribing doctor should be contacted.

**Counselling**

Pharmacist counselling is of particular importance for anti-TB medicines. Adherence is challenging given the long duration of the course, high number of pills, and potential side effects (some of which may be alarming e.g., orange or red urine and tears). Provision of information outlining what to expect when undergoing such treatment is essential to empower patients and help them to achieve treatment success. Essential medicine information that TB patients need to be made aware of includes the importance of continuing treatment even after alleviation of symptoms; the need to check for interactions before taking any other medication, given the enzyme-inducing effects of rifampicin which can alter the way other medicines work (e.g., it reduces the effectiveness of oral contraceptives); the need to limit alcohol use; the possibility that they may experience a range of side effects; and that they must report symptoms that may indicate toxicity such as changes in vision or yellow skin [29].

Alt-text: Unlabelled box

The role of registered drug shops and characterisations of ‘correct TB management’ is less well defined, and arguably more limited. For example, in Tanzania, ADDOs are legally prohibited from stocking first-line anti-TB medication [31]. Such outlets still have an important role to play in terms of case detection given the treatment seeking behaviour of patients with cough and fever, but interestingly there has been reluctance amongst other health professionals to recognise referrals from such providers (ibid).

For this paper, we reviewed the literature to examine the methods used for studying quality of care in pharmacies; the quality of TB care provided by pharmacies; and interventions to improve such care. In the three sections that follow we present our findings. The intention of this overview was not to conduct an exhaustive, systematic search of the literature; but rather, to identify key themes in the literature and provide a narrative review. Included papers were identified through the reference lists of prior, relevant reviews (including their appendices) [6,[10], [11], [12],[14], [15], [16], [17], [18]]. We included papers concerned with any of the aspects of quality laid out in Table 1, from a quantitative or qualitative perspective. Reference lists of included papers were then also screened for relevant material. We acknowledge that this approach has its limitations, and that grey literature (including TB program reports that are rarely publicly available) has not been included.

## 3 Methods for studying quality of TB care from pharmacies

Commonly used tools for measuring the quality of pharmacy care encompass surveys and medical vignettes, abstraction of information from medical records, (covert) standardised patients, exit interviews with patients, and direct observation of the patient-provider interaction. Some of these methods have been employed to measure various aspects of TB care quality in LMIC pharmacy settings.

Questionnaires have been used to measure structural aspects of care quality including staff knowledge and availability and stock management of TB medicines (e.g., [32], [33], [34]). Questionnaires have also been used to gather information on reported case management practices (ibid). Results from the latter should be interpreted with caution, however, because such reports can be subject to social desirability bias. Studies looking at a range of conditions have revealed vast discrepancies between knowledge or stated practice for hypothetical scenarios (vignettes) and actual practice for sale of medicines, referral for medical attention, history taking and provision of advice (e.g., [35,[36], [37], [38]]). This phenomenon whereby providers do not necessarily do what they know they should do has been termed the ‘know-do’ gap [39].

Three studies have used standardised patients to assess TB care, whereby people from the local community were trained to pretend to be real patients seeking care at pharmacies. They presented various pre-specified scenarios of TB and subsequently recorded the details of the encounter [30,40,41]. This method confers several advantages over alternative quality measures and has been described as the ‘gold standard’ for measuring quality [26]. It is free from the aforementioned social desirability bias associated with vignettes and the Hawthorne effect that accompanies direct observation. Further, it avoids issues of confounding that can arise due to patient and case mix [42]. In the pharmacy context, it is unsurprising that there are no studies that report using exit interviews or observation of encounters to measure TB care quality. This is because TB patients are unlikely to make up a large proportion of conditions seen at the pharmacy on a daily basis. It would therefore be logistically difficult and time consuming to collect data on this condition through such means. Pharmacies tend not to keep records of encounters with patients (asides from for the sale of restricted POMs e.g., opioids) which rules out the use of retrospective record abstraction.

Anthropologists have used ethnographic methods to better understand and describe the pharmacist-client interaction [43,44], though no such studies have reported on TB management, again likely due to its relatively uncommon presentation. However, there are examples of ethnographic work based in the community seeking to understand pharmaceutical consumption that have provided insights into care seeking and the role played by community pharmacy in TB management (e.g., [45]).

## 4 Empirical evidence on quality of TB care from pharmacies in LMICs

### 4.1 TB medicine availability

Empirical evidence on availability of anti-TB medicines from private pharmacies in LMICs is scant. It shows that availability across settings is variable, with gaps in stocking first-line anti-TB medicines ranging from a third to three quarters. A study in Cochabamba city, Bolivia, reported that 99 out of 100 sampled pharmacies were unable to completely fill a prescription for one week's supply of rifampicin 300 mg, isoniazid 150 mg, ethambutol 400 mg and pyrazinamide 500 mg [46]. Only 25% stocked at least one of the medicines. The most commonly stocked was rifampicin (23%) which was said to be used for indications other than TB. In Hanoi, Vietnam, 49% of 128 pharmacy workers said they stocked at least two first-line anti-TB medicines [41]. Surveys from Latipur, Nepal [47] and Ho Chi Minh City (HCMC), Vietnam [32], have revealed that 74% and 60% of pharmacies reported stocking anti-TB medication respectively. In Cambodia, drug availability data collected from 66 private pharmacies in 14 provinces showed that the most frequently available anti-TB medicines were pyrazinamide (71%), rifampicin (70%), streptomycin (62%), and ethambutol (56%) [34].

### 4.2 Anti-TB medicine quality

Several studies have reported on the prevalence of substandard and falsified anti-TB drugs in LMICs (e.g., [48,49]). Perhaps the largest one of its kind assessed the quality of isoniazid and rifampicin procured from private pharmacies across 19 cities in Angola, Brazil, China, DRC, Egypt, Ethiopia, Ghana, Rwanda, India, Kenya, Nigeria, Russia, Thailand, Turkey, Uganda, Tanzania, and Zambia. The authors report that of the 713 treatment packs procured, 9.1% failed basic quality testing for required levels of active pharmaceutical ingredient or disintegration [50]. Failure rates were 17%, 10% and 4% in Africa, India, and other middle-income countries respectively. These results are similar to an earlier multi-country study which tested isoniazid, rifampicin, and fixed-dose combinations (FDCs) from selected TB programmes in Colombia, Estonia, India, Latvia, Russia and Vietnam. Overall 10% of samples (4/40) were found to contain <85% of the stated content [51]. More FDCs were found to be substandard than single drug samples. Worryingly, a study from India reported that TB FDCs purchased from pharmacies all failed accelerated stability testing at 3 months having passed the initial ingredient content assays (range 90–100%) [52]. This indicates that under common Indian climatic conditions, TB medicines being purchased may be unstable.

### 4.3 Knowledge of TB amongst pharmacy providers

Across a range of studies knowledge was variable, with some indicators revealing poor results. Several studies have collected data on knowledge pertaining to the activities of the national control programme. For example, a study of 300 randomly selected pharmacies in Tamil Nadu, India, reported that all pharmacists were aware of the availability of free anti-TB medicines from government facilities; but fewer (15%) had heard of the national TB programme and only 5% knew about the DOTS strategy [53]. Figures differed in other settings with two thirds of pharmacies in the Vietnamese capital, Ho Chi Minh City (HCMC), stating they were aware of the national programme; in another large Vietnamese city, Hanoi, 27% of study pharmacies were aware that the national programme provided free treatment; and in Nigeria, a study of PMVs revealed that over half had never heard of DOTS [32,33]. Studies have also sought to measure knowledge of TB management and symptoms; results have tended to be disappointing. In Nepal, 14% of study pharmacy workers believed TB treatment duration was less than four months and only 1/50 knew the correct regimen [54]; in Vietnam, when presented with a case description of a patient with cough for 4 weeks and fever, only 18% of participants in HCMC and 42% in Hanoi mentioned TB as a potential diagnosis [32,41]; in Nigeria around half of PMVs did not know the cause of TB [33].

### 4.4 Reported management practices for TB patients

We are only aware of a handful studies that have explored self-reported management practices for TB patients among pharmacy providers. A high proportion (88%) of pharmacies in HCMC said that a patient with pulmonary TB required medical attention to ascertain a diagnosis [32]. Over half of these same participants reported selling anti-TB drugs sometimes or often, and of those, 24% reported selling such drugs without a prescription. Of 388 PMV study participants in Nigeria, fewer than 10% reported sending patients with prolonged cough or suspected TB for a laboratory test, and just over half (57%) reported referring TB cases to a higher facility [33].

### 4.5 Actual management practices for TB patients

Standardised patients have been used to show how TB suspects are managed in practice, in both India [30,40] and Vietnam [41]. These studies have used varying case presentations (Table 1). In Vietnam, we are not aware of any specific guidelines for the management of TB patients by pharmacists. As per Box 1, we would expect patients presenting with TB symptoms to be referred. The study reports that fewer than half of 126 pharmacies [46] referred the SP for diagnosis; only 9% referred to a designated TB facility; 53% sold medicines to the SP; and 41% sold an antibiotic [41].

The SP studies in India benchmarked the management of these patients against the recommendations of the RNTCP and the Indian Pharmaceutical Association [28]. ‘Correct’ management in both studies was defined as referral to a TB clinic/DOTS centre or heath care provider without dispensing any antibiotics or steroids (which were deemed to be harmful). Depending on the case presentation, correct management ranged from 13% to 62% and dispensing of an antibiotic from 16% to 37% [55]. In a pooled analysis of results across the three Indian cities from both studies, the authors reported that behaviour improved markedly as the certainty of the diagnosis was more apparent (ibid). For example, those presenting with cough and fever, for which there are several differential diagnoses, were only managed correctly by 13% of providers. This increased to 45% when the SP mentioned contact with a relative with a TB diagnosis; and to 62% when they presented a confirmed positive sputum test. Miller and Goodman [40] also reported on history taking and advice provision. They found that less than a quarter of providers asked the SP any questions to determine a diagnosis (a necessity given that the symptoms of cough and fever are non-specific); fewer than 2% advised that TB treatment was available free of charge from government facilities. Encouragingly, none of the providers in either India or Vietnam sold first line anti-TB drugs to the SPs.

## 5 Interventions to improve TB care from pharmacies in LMICs

In its 2006 document ‘Engaging all health care providers in TB control’ the WHO outlined the importance of engaging pharmacists and drug outlets in national TB control efforts and provided guidance on how to implement public-private mix (PPM) approaches [8]. Konduri and colleagues conducted a comprehensive review of ‘engagement of the private pharmaceutical sector for TB control’ and identified 52 interventions involving retail drug outlets [10]. However, the majority of these interventions were identified from conference abstracts, which provide limited detail. 15 provided data on the number or percentage of referrals of presumptive TB cases or resulting positive cases. These interventions (carried out between 2003 and 2014) involved between 60 and 683 retail drug outlets; referrals ranged between 0.25 and 9 per retail outlet; of referrals, the percentage screened ranged from 27% and 91%; and the number of smear positive cases ranged from 3 to 395 (ibid). This review concluded that, to date, efforts to engage pharmacies have been limited. In this section, we focus on seven interventions (for which full research papers are available) that have sought to address at least one aspect of quality of care laid out in Table 1 (see Table 3 for details). All were carried out in conjunction with the national TB programme of the study country and are classified as PPM projects [56], [57], [58], [59], [60], [61], [62], [63].

**Table 3:** Interventions to improve management of TB in the pharmacy setting in LMICs.

Author	Country and programme	Details of intervention	Key findings/outcomes
Bell et al. [56]	Phnom Penh, Cambodia National center for Tuberculosis and Leprosy Control public/private mix TB Referral Programme	- 170 private pharmacies. - Clients with TB symptoms are referred by pharmacy to public sector DOTS clinics. - 3-day training for pharmacy staff and visits to DOTS clinics.	During previous 3 months: - One third of the pharmacies reported referring one or more clients. - The 170 pharmacies referred a total of 125 clients. - 96% stated they always referred all clients with TB symptoms to DOTS clinics.
Colvin et al. [57]	Kisarawe district, Tanzania National Tuberculosis and Leprosy Programme, PATH, and USAID.	- 15 pharmacists (and 15 traditional healers) received 2 days training. - Pharmacies given referral slips and registers to track referrals to DOTS, and directory of DOTS facilities.	- Between 2009 and 2011 smear-positive TB case notification increased from 28 to 47/100,000 - Pharmacies referred 434 people to diagnostic facilities. 97% acted on the referral, and of these, 25% were diagnosed with TB. - New TB case notifications (in the study district) referred through the network ranged from 38% to 70%.
Gharhat et al. [59]	Mumbai, India Mumbai District Tuberculosis Control Society, colleges of pharmacy, professional associations of pharmacists and physicians.	- 119 pharmacist - 2 interactive workshops - Advised to refer patients with suspected TB and to counsel patients prescribed anti-TB medicines	- Anecdotally, participation in the workshops was associated with a high degree of professional satisfaction. - No measurement of pharmacist performance post training.
Lonnroth et al. [61]; Quy, et al. [63]	Ho Chi Minh City, Vietnam	- 150 pharmacies trained according to NTP guidelines - New referral and recording system. - Clients with TB symptoms referred for sputum smear microscopy at a District TB Unit. - US $1 for each sputum-positive detected case	- 39% referred at least one client to a TB - 310 TB suspects were referred during first 9-month monitoring (only 28% went for testing). - An additional 63 patients were referred and tested in 2nd follow-up. - 7% of the 149 patients tested were sputum-positive (accounting for 1.6% of cases detected in the intervention (others resulted from GP and physician referrals).
Mitchell et al. [62]	Santo Domingo, Dominican Republic	- Intervention aimed at pharmacies and local grocery stores - Components of intervention involved a 1 h educational workshop and a motivational ‘detailing visit’ - Participants were invited to sign a pledge and receive a certificate of recognition. - SPs (reporting a set of chronic TB symptoms) were sent 3–6 weeks before the interventions began and again 2–6 weeks afterwards.	- Pharmacies exposed to the intervention improved by 2.12 points (score based on TB behaviours e.g., recognition of symptoms) on average compared with an improvement of 0.9 in the comparison group (*p* = =0.06) - Half of intervention pharmacies referred SPs directly to the national TB program vs. 18.2% of the control group. - After intervention attempts to sell a medicine including antibiotics without a prescription (e.g., amoxicillin, cephalosporin, rifampicin) fell from 38% of pharmacies to none. - At 6 months follow-up, 33% of pharmacies referred 70 TB suspects of which 7 cases (10%) resulted in a smear positive diagnosis. - At 2 year follow-up, detection of new smear positive cases averaged 150 per quarter vs. 67 per quarter in the pre-intervention period.
Daftary et al. [58]	Patna, India Intervention nested into Universal Access to TB Care (a PPM programme between Bihar state government and PPIA World Health Partners).	- Broader PPM programme involved 554 pharmacies in standardised TB management plus incentive of US $0.75 for each completed referral. - Intervention (105 pharmacies) had 5 additional components: interactive training workshops; referral of TB suspects for chest radiograph and doctor consultation; financial incentives for referral completion, chest radiograph and positive TB diagnosis ($0.75, $1.50, and $3 respectively), text message reminders and field support.	- 81% of pharmacy providers actively participated in the pilot vs. 16% in original PPM programme. - Rate of registration of patients with TB symptoms and positive TB diagnoses were 62 and 25 times higher respectively in the intervention group. - Microbiological testing and test confirmation was also significantly higher in the intervention group. - 240 additional cases were attributed to the intervention with a cost per case notified of US$100.
Lambert et al. [60]	Cochabamba, Bolivia NTP and local pharmacists association (ASPROFAR)	- A two stage intervention - Phase 1: 170 pharmacists attended a general meeting and local pharmacists association issued a recommendation to members to stop selling anti-TB medicines and refer clients seeking to public services. - Phase 2: 70 pharmacies referred clients with chronic cough to NTP (via referral slip).	- After phase 1, the proportion of pharmacies selling TB drugs decreased (rifampicin: 23–11.5%; isoniazid: 16–3.1%; *P* < 0.001) and the proportion of pharmacies referring to the NTP clients seeking TB drugs increased (22–58%; *P* < 0.0001). - In phase two, 38% referred a total of 41 clients for screening in the NTP; 11 of 41 (27%) were screened and of these, 3 (27%), were diagnosed with smear-positive TB.

All interventions aimed to improve the management of TB patients by participating pharmacies through training of pharmacy staff to screen patients and appropriately refer TB suspects, with the ultimate aim of improving case detection. Most training focussed on symptoms of TB and identifying patients to refer for testing. A minority of interventions also focussed on rational medicine use and antibiotic stewardship [58,59]. One study mentioned that the training covered counselling for patients prescribed anti-TB medication [59]. In addition to improving case detection efforts, one intervention additionally sought to stop pharmacies from selling anti-TB medicines [60]. Two interventions in India and Vietnam incorporated financial incentives for pharmacy referrals [58,63].

Most programmes had a relatively low engagement with around 30–40% of pharmacies actively participating in the referral process; Daftary and colleagues [58] are the exception, reporting 81% active participation in Patna, India. Workload, patient demand for OTC medicines, doctor fees, programme paperwork, fear of losing patients to other pharmacies (and hence the opportunity to sell medicines such as antibiotics and cough suppressants), and concern that patients would criticise the pharmacist were they to receive a negative TB diagnosis all negatively affected engagement [58,61]. Across the studies, of patients referred by pharmacies, positive TB diagnoses ranged from 7 to 27% of patients tested. In India, an intervention which added several components to a broader PPM programme, reported a TB diagnosis rate that was 25 times higher than the standard programme alone, indicating that a multi-faceted approach including financial incentives, regular SMS reminders, and regular supervision and monitoring could have a key role in maintaining high levels of pharmacy engagement [58]. One study using SPs to assess the effects of the intervention reported reductions in both antibiotic and cold remedy sales, alongside improved detection rates [62]. Finally, a lack of cost data is evident, with only one study considering the cost-effectiveness of its case finding efforts.

## 6 Discussion and future directions

In this paper we mapped out what is expected of pharmacies in relation to quality TB care and then examined the evidence to shed light on the degree to which this is occurring. The expectations of pharmacists included matters pertaining to accurate dispensing of medicines but also encompassed wider issues such as case management. This is in line with global thinking regarding the role of community pharmacists which has seen a shift from solely retailing of products to taking on a substantial public health role comprising promotion, prevention, and disease management [19]. Mossialos and colleagues argue that no country can boast the latter (ibid). In LMICs there is an increasing acknowledgement, and advocacy from both national and global pharmacy bodies that pharmacists in these settings could (and should) provide a comprehensive pharmaceutical service. This is evidenced, for example, by the International Pharmaceutical Federation's ‘Good Pharmacy Practice (GPP) in developing countries’ guide [20]. Further illustrations of this shift include Indonesia's ‘Pharmaceutical Services Guidelines’ in ‘apoteks’ (pharmacies) [21] and the Pharmacy Council of India's 2015 ‘Pharmacy Practice Regulations’. The latter clearly lays out a code of ethics, and the duties and responsibilities of a pharmacist (which include promoting rational drug use, patient counselling, management of minor ailments, and public health duties) [22].

While the empirical literature on quality of TB care from pharmacies in LMICs is relatively sparse, the quality challenges are clearly evident, relating to anti-TB medicine availability, quality of anti-TB medicines, and pharmacy worker's knowledge. Further, management practices suffer from a lack of questioning of symptomatic patients to ascertain whether they require testing, lack of referral of TB suspects, sale of unnecessary and harmful medicines, sale of anti-TB medication OTC, and lack of counselling. These findings are very much in keeping with the shortfalls in pharmacy practice identified for other diseases [11,12].

Given that it is 13 years since the WHO actively encouraged countries to engage pharmacists and drug shops into national TB programmes [8], the number of published interventions in this area is strikingly few (we acknowledge that there are a number of interventions lacking published findings). Of the interventions scrutinised by this paper (Table 3), participants found the interventions to be acceptable and many reported professional satisfaction from their involvement. Whilst active participation in these programmes appears to have been low, with the right set of incentives and support, high participation levels have been achieved and in one study case-detection through pharmacies was shown to be cost-effective [58].

All these interventions have explicitly focussed on engaging pharmacies in the national TB programme to improve case detection. Whilst appropriate referrals and hence improved case detection represents an important aspect of quality, the narrative of these papers appears to lack discussion of other dimensions of quality of care. It is clear that, thus far, a focus on quality has been a ‘missing ingredient’ in efforts to harness pharmacies to improve TB control [13]. Future interventions would do well to tackle the seemingly neglected areas of OTC prescribing of unnecessary and harmful medicines, and measures to improve patient counselling to accompany the sale of anti-TB medicines. The implemented interventions have generally shown a reasonable degree of success but achieving scale remains a challenge, a challenge also identified with engagement of other for-profit providers [64]. An analysis of the problems in engaging private providers more broadly highlighted a number of barriers to reaching scale in PPM programmes. These include, amongst others, a bias towards public provision, lack of funding, a lack of understanding of private healthcare markets, high level of fragmentation in these markets, market incentives favouring poor quality care, and competing priorities [6]. Building a strong evidence base, advocating for enhanced political commitment and funding, utilisation of digital technology, setting ambitious PPM targets, and monitoring the progress of PPM initiatives may help to overcome some of these obstacles [64].

It was noticeable that none of the interventions included a regulatory component, although regulatory systems exist with mandates for pharmacy in all countries. Lack of regulatory oversight is a clear problem in the pharmacy retail sector in many LMICs and an area that warrants attention (both generally and in relation to TB care). Regulatory interventions have shown promising improvements in pharmacy service quality in the past [65,66]. Whilst studies reported that sales of anti-TB medicines OTC were commonplace in some countries e.g., Vietnam [32]; in other countries, such as India, this was reported to be rare (despite being very common other antibiotics) (e.g., [30,55]). A detailed examination of regulatory policies surrounding anti-TB medicine may provide useful cross-country learning experiences. For example, other countries could explore the possibility of initiating hierarchies of drug control in the way that India has instigated the H1 drug schedule which includes more restrictions and stricter penalties for selling anti-TB medicines in a bid to halt the emergence of resistant antibiotic strains.

This paper has also highlighted the lack of explicit global-level guidelines aimed at pharmacies, outlining how they should manage TB patients. This has similarly been emphasised as a major gap for the management of other conditions that present at the pharmacy, such as childhood diarrhoea [40]. A concise handbook aimed at LMIC pharmacies outlining the key management ‘dos and don'ts’ for conditions of high public health importance would be a key contribution from the global health community. Such an endeavour could draw on guidelines previously produced by local pharmacy associations (e.g., the India Pharmaceutical Association's guidance on TB management [28]). A key first step would be the identification of all such existing guidance for a range of conditions (which is often not publicly available or only available in a local language). Organisations such as FIP and the WHO (specifically the Department of Essential Medicines and Health Products), which have a strong presence and legitimacy in the global pharmacy space, would be well-placed to spearhead such an initiative. Appropriate training should also be incorporated into pharmacy pre-service education. Additionally, the role of non-pharmacist run pharmacies and drug shops requires clarification.

Looking forward, technological innovations could further extend the role of pharmacies in TB care. Glaze and Rowe have suggested that pharmacists in the US are well placed to administer a purified protein derivative skin test, read the results, and provide education to the patient [67]. Moulding proposes that private doctors in LMICs could prescribe ‘monitored self-administered treatment’, whereby pharmacies would fill and dispense medication monitors (as opposed to anti-TB medication either loose or in its original packaging) in order to improve adherence in the private sector [68].

While many approaches are possible, the current reality of pharmacy care for TB patients remains substandard. Given the importance of pharmacies in LMICs as an early point of contact for TB patients, there remains a critical agenda of work to ensure the potential benefits for TB control can be maximised. Addressing quality of care at the pharmacy level, will require the participation and collaboration of a wide range of stakeholders, going well beyond the pharmacies themselves. This complex landscape involves organisations at the global level, such as WHO, FIP, and the STOP TB programme; and at the country level, national TB programmes, regulatory bodies, pharmacy associations, pharmacy colleges and ministries of health. Given that in the TB context, community pharmacy sits at the intersection between policies for TB and policies that focus on pharmacy more generally, these two groups of stakeholders will need to come together to effect change.

## Ethical statement

This paper is based solely on literature within the public domain.

## Funding

RM acknowledges post-doctoral funding from the Economic and Social Research Council, UK. Grant reference ES/T006854/1.

## Declaration of Competing Interest

We declare that we have no conflict of interest.
